# Flow-Volume Parameters in COPD Related to Extended Measurements of Lung Volume, Diffusion, and Resistance

**DOI:** 10.1155/2013/782052

**Published:** 2013-06-13

**Authors:** Linnea Jarenbäck, Jaro Ankerst, Leif Bjermer, Ellen Tufvesson

**Affiliations:** Department of Clinical Sciences, Respiratory Medicine and Allergology, Lund University, 221 84 Lund, Sweden

## Abstract

Classification of COPD into different GOLD stages is based on forced expiratory volume in 1 s (FEV_1_) and forced vital capacity (FVC) but has shown to be of limited value. The aim of the study was to relate spirometry values to more advanced measures of lung function in COPD patients compared to healthy smokers. The lung function of 65 COPD patients and 34 healthy smokers was investigated using flow-volume spirometry, body plethysmography, single breath helium dilution with CO-diffusion, and impulse oscillometry. All lung function parameters, measured by body plethysmography, CO-diffusion, and impulse oscillometry, were increasingly affected through increasing GOLD stage but did not correlate with FEV_1_ within any GOLD stage. In contrast, they correlated fairly well with FVC%p, FEV_1_/FVC, and inspiratory capacity. Residual volume (RV) measured by body plethysmography increased through GOLD stages, while RV measured by helium dilution decreased. The difference between these RV provided valuable additional information and correlated with most other lung function parameters measured by body plethysmography and CO-diffusion. Airway resistance measured by body plethysmography and impulse oscillometry correlated within COPD stages. Different lung function parameters are of importance in COPD, and a thorough patient characterization is important to understand the disease.

## 1. Introduction

Spirometry and body plethysmography are the most commonly used methods to diagnose, characterize, and assess chronic pulmonary obstructive disease (COPD). The global initiative of obstructive lung diseases (GOLD) classification of COPD [1] is acknowledged around the globe and is recommended both by the American Thoracic Society and the European Respiratory Society. It has long been based on spirometry and health status alone. However, a new version from 2011 proposes the importance of considering exacerbation frequency and assessing the severity of breathlessness, using the modified Medical Research Council Questionnaire (mMRC), in the classification of COPD. For practical purposes, flow-volume spirometry is used to characterize lung function in COPD patients. It is easily used, and the measurements derive reproducible data. Forced expiratory volume in 1 s (FEV_1_) is most commonly used but is of limited value in relation to functional ability and quality of life when used alone [2, 3]. On the other hand, spirometry also provides data of forced vital capacity (FVC) and inspiratory capacity (IC) which are the tools of choice for most population surveys.

It has long been known that spirometry measures mostly the proximal parts of the airway, while COPD is mostly a disease of the distal airways [4]. Akamatsu et al. screened patients from a nonrespiratory section of the hospital, including smokers, former smokers, and never-smokers [5]. They found that 25 out of 288 patients had COPD according to the GOLD standard (21 patients GOLD1, 4 patients GOLD2), but 52% of these patients still claimed to have no respiratory symptoms at all. This suggests that the symptoms of COPD can develop later in the disease stage. It is important to diagnose the patients at an early stage since the disease is progressive and irreversible. Since no treatment is available to stop the progression in the early stage, it is of great importance to identify patients in this stage to evaluate novel therapies for disease progression.

It is therefore important to use plausible lung function measurements for a satisfactory diagnosis and monitoring of COPD. Body plethysmography and single breath helium dilution with carbon monoxide- (CO-) diffusion are two commonly used techniques to evaluate lung volumes in order to look at hyperinflation that is not reflected by spirometry. However, the helium dilution method is known to underestimate lung volumes, while body plethysmography measures increased lung volumes in obstructive patients [6]. After administration of tiotropium for two weeks in obstructive patients with hyperinflation, lung volumes such as residual volume (RV) and functional residual capacity (FRC) measured with body plethysmography decreased, while RV and FRC measured by helium dilution method increased [7].

Impulse oscillometry (IOS) can detect distal airway malfunctions that are not measured with normal spirometry. COPD patients have a higher total resistance (R5), and peripheral resistance (R5–R20), and a more negative reactance at 5 Hz (X5) than healthy never-smokers [8]. Increased effect on R5, R5–R20, and X5 was seen with increased disease severity. However, none of the IOS parameters could separate healthy never-smokers from GOLD1 [8]. Interestingly, subgroups of COPD patients showed normal IOS values, as some patients with low reactance area (AX) displayed low FEV_1_, and patients with abnormal R5 showed less emphysema [9]. Several studies have shown a correlation between several IOS parameters and FEV_1_ [8, 10, 11], CT scans, dyspnea, and health status [12]. Frantz et al. recently showed that patients with self-reported chronic bronchitis, emphysema, or COPD have higher resistance and lower reactance than patients without self-reported disease independent of spirometry-based diagnosis [13]. This suggests that IOS could be used to detect pathological changes in COPD earlier than spirometry. In contrast, it has been shown that commonly used pulmonary function tests were more sensitive in detecting COPD than was IOS but had the same specificity in excluding COPD [14].

The aim of the present study was to relate established flow-volume spirometry values to other more advanced measures of lung function using body plethysmography, single breath helium dilution with CO-diffusion and IOS in COPD patients in different stages, and healthy smokers that have not developed COPD. A secondary aim was to evaluate better characterization of lung function impairment of importance in different degrees of COPD. We hope to expand characterization of COPD patients using other parameters than from normally used flow-volume measurements to get an extended picture of the lung physiology in different COPD phenotypes.

## 2. Methods

### 2.1. Subjects

Ninety-nine volunteers were screened with spirometry; 65 were classified as COPD patients (FEV_1_/FVC < 0.7) and 34 as healthy smokers (FEV_1_ ≥ 80%, FEV_1_/FVC ≥ 0.7) (Table 1). The COPD patients were diagnosed and categorized into GOLD stages according to GOLD standards (http://www.goldcopd.org/ version 2011 [1]). Thirteen GOLD1 (FEV_1_ ≥ 80% of predicted normal), 22 GOLD2 (50 ≥ FEV_1_ < 80% of predicted normal), 15 GOLD3 (30 ≥ FEV_1_ < 50% of predicted normal) and 15 GOLD4 (FEV_1_ < 30% of predicted normal) were included. Study participants had no history of lung cancer, asthma,or cardiorespiratory diseases and had a history as smokers or former smokers with ≥15 pack years. Neither exacerbation nor respiratory infection was allowed within the last 3 weeks. All lung function measurements were done after receiving 400 *μ*g short-acting beta-2 agonist (salbutamol, Buventol Easyhaler) according to the GOLD classification system. Three patients with GOLD3 and eight patients with GOLD4 had also inhaled long-acting muscarinic antagonists (18 *μ*g tiotropium, Spiriva).

### 2.2. Study Design

The study was approved by the Regional Ethical Review Board in Lund (431/2008), and all study participants signed written informed consent. A physical examination was performed before the start of the study. All subjects performed IOS (Jaeger MasterScreen, Erich Jaeger GmbH, Würzburg, Germany), body plethysmography together with flow-volume spirometry (MasterScreen Body Jaeger) and single breath helium dilution with CO-diffusion test (MasterScreen Diffusion Jaeger) in given order. FEV_1_ and FVC were measured using established flow-volume spirometry, and FEV_1_/FVC was calculated. From body plethysmography (BP) inspiratory resistance (*R*_in_), expiratory resistance (*R*_ex_), IC, RV_BP_, total lung capacity (TLC)_BP_, and FRC_BP_ were recorded. The technique of single breath helium dilution with CO-diffusion tests (SB) estimates lung volumes, such as RV_SB_, TLC_SB_, and FRC_SB_, diffusing capacity of the lung for carbon monoxide (DLCO) and alveolar volume (VA) was measured, and DLCO/VA was calculated. Resistance at 5 HZ (R5; total resistance) and 20 Hz (R20; central resistance), Resonance frequency (Fres), Reactance at 5 Hz (X5), and Reactance area (AX) were measured by IOS, and R5–R20 (peripheral resistance) was subsequently calculated. All lung function measurements were made according to ERS/ATS standardizations [15–17]. Reference values established by Crapo were used [18]. Information about COPD symptoms was documented in a self-filled in Clinical COPD Questionnaire (CCQ) [19].

### 2.3. Statistics

Nonparametric unpaired data were analyzed first using the Kruskal-Wallis test for trend analyses between several groups and thereafter the Mann-Whitney test between two groups (with correction for ties). Paired data were analyzed using the Wilcoxon test. Correlations were analyzed using Spearman's nonparametric correlation test. All statistical analyses were done using SPSS 20.0 for Windows (SPSS, Inc., Chicago, IL, USA), and a *P* value <0.05 was considered significant. All data were presented as median (interquartile range).

## 3. Results

### 3.1. Patient Characteristics

There were no significant differences in sex or body mass index between healthy smokers and COPD patients (Table 1). All subjects had matched age (except for patients with GOLD2 that were younger than healthy controls), and pack years (except for patients with GOLD3 who had more pack years). CCQ value increased with increasing GOLD stage and was higher in GOLD stage 2–4 compared to healthy smokers (Table 1). One healthy smoker, three patients with GOLD2 and one patient with GOLD4 had low levels of alpha_1_ antitrypsin (<0.86 g/L for men and <0.94 g/L for women). According to patient classification, FEV_1_/FVC differed significantly between healthy smokers and GOLD1 but also continued to decrease with increasing GOLD stage. An interesting increase in FVC%p was seen in GOLD1 compared to healthy smokers, and thereafter FVC%p decreased with increasing GOLD stage.

### 3.2. Body Plethysmography

**Figure 1:**
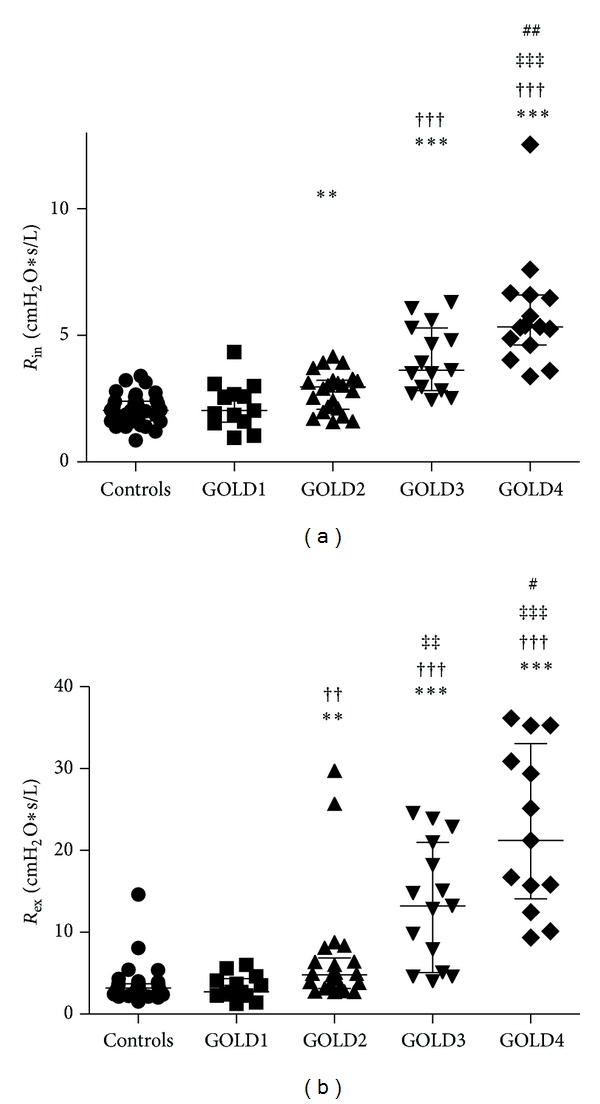
*R*_in_ (a) and *R*_ex_ (b) measured by body plethysmography in controls (healthy smokers) and COPD patients with GOLD stage 1–4. *Significant difference compared to healthy smokers, ^†^significant difference compared to GOLD1, ^‡^significant difference compared to GOLD2, ^#^significant difference compared to GOLD3, one symbol flagging *P* < 0.05, two symbols flagging *P* < 0.01, and three symbols flagging *P* < 0.001. Data are presented as individual dots together with median with interquartile range.

The Kruskal-Wallis test showed an overall increasing trend among the groups for both *R*_in_ and *R*_ex_ (*P* < 0.001). Both the *R*_in_ and the *R*_ex_ measured with body plethysmography were increased in GOLD2–4 compared to healthy smokers (Figures 1(a) and 1(b)). IC was decreased, but only in later stages of the disease (GOLD3-4) (Table 2).

### 3.3. Increase in Lung Volume Measured by Body Plethysmography and Single Breath Helium Dilution with CO-Diffusion Already in GOLD1

**Figure 2:**
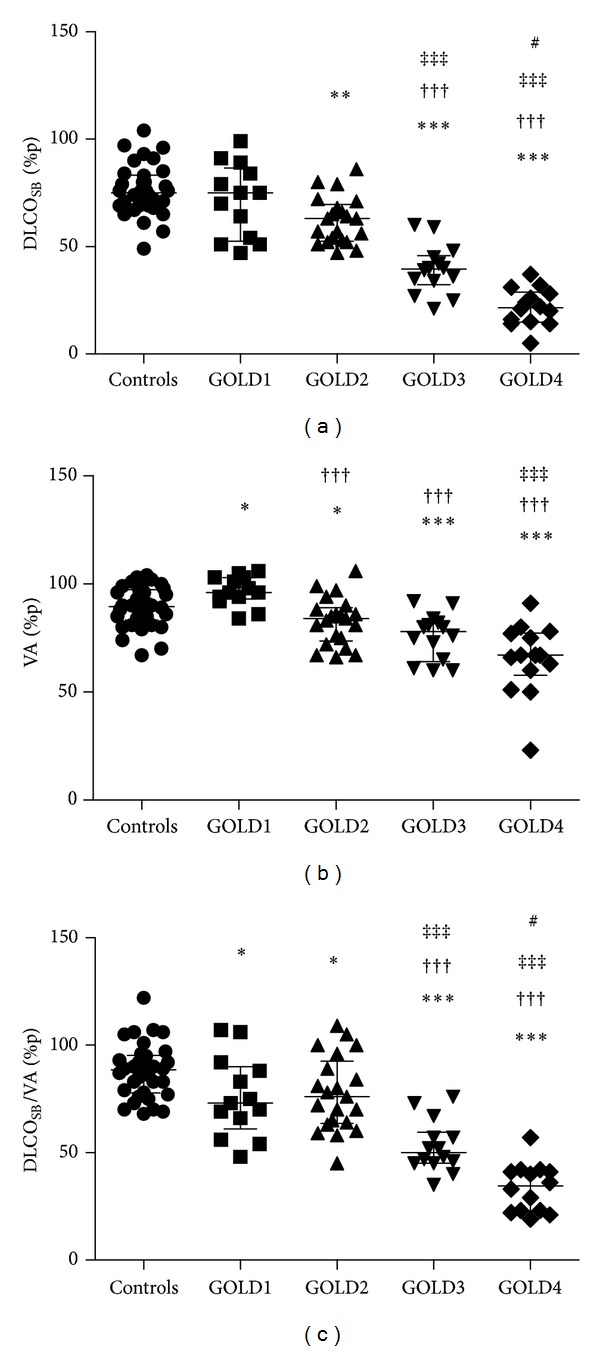
DLCO_SB_%p (a), VA%p (b), and DLCO_SB_/VA%p (c) measured by single breath helium dilution with CO-diffusion in controls (healthy smokers) and COPD patients with GOLD stage 1–4. *Significant difference compared to healthy smokers, ^†^significant difference compared to GOLD1, ^‡^significant difference compared to GOLD2, ^#^significant difference compared to GOLD3, one symbol flagging *P* < 0.05, two symbols flagging *P* < 0.01, and three symbols flagging *P* < 0.001. Data are presented as individual dots together with median with interquartile range.

An increasing trend among all the groups was seen for TLC%p_BP_ (*P* < 0.01), RV%p_BP_ (*P* < 0.001), and for VA%p_SB_ (*P* < 0.001) using the Kruskal-Wallis test. Interestingly, both TLC%p_BP_ and FRC%p_BP_ measured with body plethysmography were already significantly increased in GOLD1 (Table 2). In conjunction with this, the alveolar volume (VA%p) measured by single breath helium dilution with CO-diffusion was increased in GOLD1 and decreased in GOLD2–4 compared to healthy smokers (Figure 2). 

### 3.4. Diffusing Capacity Decreased with Increasing GOLD Stage

An overall difference between the groups regarding diffusion capacity was detected using Kruskal-Wallis. The diffusing capacity (DLCO%p) was decreased in GOLD2–4 compared to healthy smokers. When divided by the alveolar volume (DLCO/VA) a decrease was already seen from GOLD1, due to the early increase in VA%p seen in GOLD1, and extended to GOLD4 (Figure 2, Table 2).

### 3.5. Difference in RV and TLC Measured by Body Plethysmography and Single Breath Helium Dilution with CO-Diffusion

**Figure 3:**
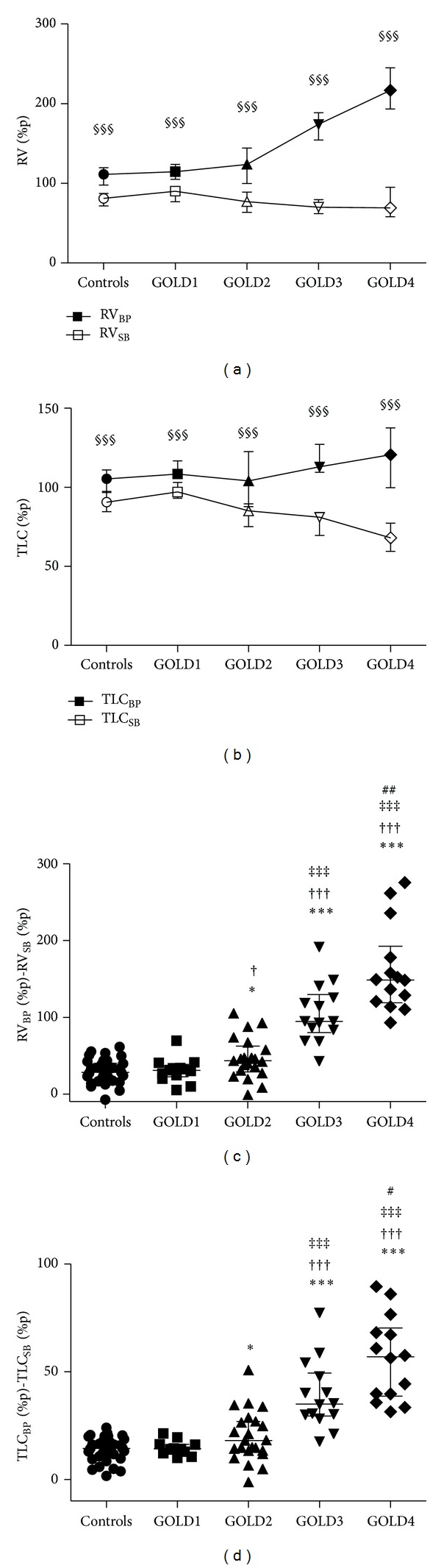
RV% (a) and TLC%p (b) measured by body plethysmography and single breath helium dilution with CO-diffusion. Difference in RV% (RV%p_BP−SB_) (c) and TLC% (TLC%p_BP−SB_) (d) measured by body plethysmography and single breath helium dilution with CO-diffusion in controls (healthy smokers) and COPD patients with GOLD stage 1–4. *Significant difference compared to healthy smokers, ^†^significant difference compared to GOLD1, ^‡^significant difference compared to GOLD2, ^#^significant difference compared to GOLD3; ^§^significant difference between measurement from body plethysmography compared to single breath helium dilution with CO-diffusion, one symbol flagging *P* < 0.05, two symbols flagging *P* < 0.01, and three symbols flagging *P* < 0.001. Data are presented as median (IQR) in (a)-(b) and individual dots together with median with interquartile range (c)-(d).

RV measured with body plethysmography (RV%p_BP_) was increased only in later stages of the disease (GOLD3-4, Table 2). In contrast, a parallel decrease in RV measured by single breath helium dilution with CO-diffusion (RV%p_SB_) was seen (Figure 3(a)) and decreased by advancing GOLD stages. This indicates increased air trapping. To pronounce the outcome on individuals' RV, a difference in RV measured with body plethysmography and by single breath helium dilution with CO-diffusion was calculated (RV%p_BP−SB_). A clear increasing pattern in RV%p_BP−SB_ was seen with increasing GOLD stage (Figure 3(c)) already from GOLD2.

A similar pattern was seen for TLC, but not as pronounced as for RV. An increase in TLC%p_BP_ was seen in GOLD3-4, together with a decrease in TLC%p_SB_ (Figure 3(b)) in GOLD2–4. Individual differences in TLC%p (TLC%p_BP−SB_) show a clear increasing pattern through the GOLD stages already from GOLD2 (Figure 3(d)).

### 3.6. IOS Parameters Increased with Increasing GOLD Stage

**Figure 4:**
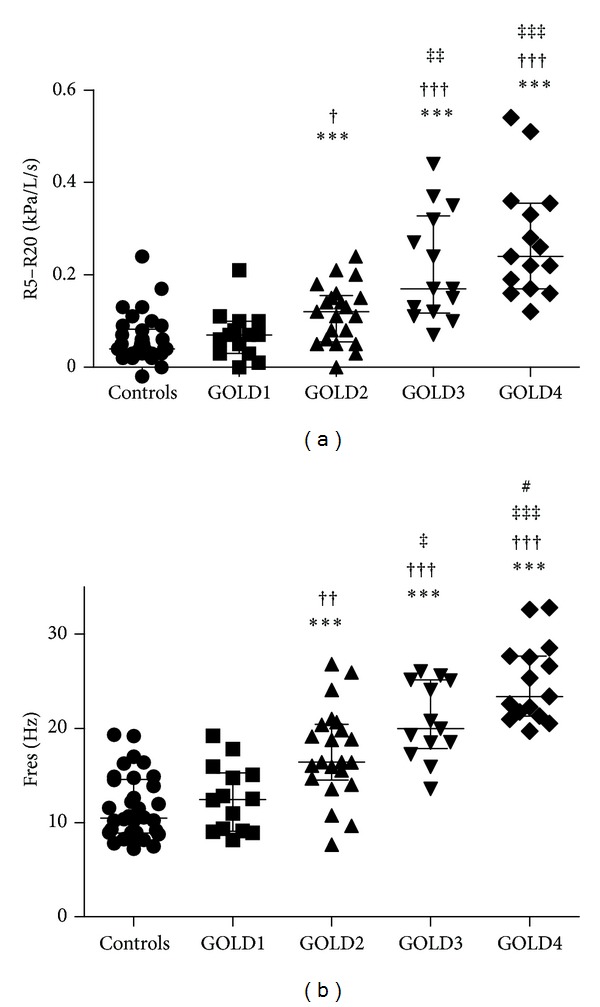
R5–R20 (a) and Fres (b) measured by impulse oscillometry in controls (healthy smokers) and COPD patients with GOLD stage 1–4. *significant difference compared to healthy smokers, ^†^significant difference compared to GOLD1, ^‡^significant difference compared to GOLD2, ^#^significant difference compared to GOLD3, one symbol flagging *P* < 0.05, two symbols flagging *P* < 0.01 and three symbols flagging *P* < 0.001. Data presented as individual dots together with median with interquartile range.

Trends of difference between groups were detected by the Kruskal-Wallis test, and all IOS parameters showed similar patterns, with no difference between healthy smokers and GOLD1, but increasing significantly from GOLD2 (except for R20) to GOLD4 (Figure 4, Table 3).

### 3.7. Established FEV_1_%p Did Not Correlate with Extended Lung Volume and Diffusing Capacity Measurements

Due to an increasing effect in all lung function parameters with increasing GOLD stage, there was also an evident overall correlation between all lung function parameters within all subjects (data not shown). When correlating the conventionally used parameter FEV_1_%p within each GOLD stage, no correlation was seen with any parameters measured by body plethysmography, single breath helium dilution with CO-diffusion, or IOS. Correlations to a subset of the parameters (that differ most pronouncedly between the different GOLD stages) are shown in Table 4. On the other hand, FVC%p and FEV_1_/FVC correlated significantly with some lung function parameters, such as RV%p_BP−SB_ and TLC%p_BP−SB_.

The difference in RV%p (RV%p_BP−SB_) strongly correlated with several lung volume and diffusing capacity parameters, such as IC %p, FRC%p, TLC%p, TLC%p_BP−SB_, and DLCO/VA%p, within most GOLD stages. The difference in TLC%p (TLC%p_BP−SB_) correlated in a similar way to IC %p, FRC%p, RV%p, RV%p_BP−SB_, and DLCO/VA%p.

### 3.8. Correlations between Parameters of Resistance Measured by Body Plethysmography and IOS, but Not to Lung Volume or Diffusing Capacity Parameters

An interesting finding was that resistance parameters measured by body plethysmography (*R*_in_ and *R*_ex_) correlated significantly with several resistance and reactance parameters measured by IOS. *R*_in_ and *R*_ex_ correlated with R5, R20, R5–R20, and Fres (Table 4) in most GOLD stages (and most pronouncedly in early GOLD stages) and AX and X5 in all GOLD stages. However, neither resistance parameters measured by body plethysmography nor IOS (except for R5–R20 in GOLD4) correlated with lung volume or diffusion parameters in any GOLD stage.

### 3.9. Dyspnea Did Not Correlate to Lung Function Parameters in Different GOLD Stages

The CCQ score increased with increasing GOLD stage (Table 1), and hence there was an apparent overall correlation with all lung function parameters. However, within the different GOLD stages there was no correlation between the CCQ score and any lung function parameter measured with spirometry, body plethysmography, and single breath helium dilution with CO-diffusion or IOS. 

## 4. Discussion

The main finding of this study was that established flow-volume parameters, such as FEV_1_, did not correlate with advanced measurements of lung volume, diffusing capacity, and resistance. This illustrates that FEV_1_ alone is not a good parameter when used for diagnosis and monitoring of COPD since it does not represent the whole picture of the disease. An interesting parameter was, however, the difference in RV%p measured with body plethysmography and single breath helium dilution with CO-diffusion. The RV%p_BP_ measured with body plethysmography was increased in parallel with a decrease in RV%p_SB_ measured with single breath helium dilution with CO-diffusion with increasing COPD severity. When using the difference between the two RV (RV%p_BP−SB_), a clearer and more pronounced pattern appeared, and the effect on lung volume becomes apparent in an earlier disease stage. This provides a good opportunity to measure air trapping and degree of hyperinflation. RV%p_BP−SB_ also correlated with several lung volume parameters, such as IC%p, FRC%p, TLC%p, and DLCO/VA%p, showing this to be an important factor in COPD characterization. A similar parameter, with similar characteristics, was the difference between TLC%p measured with body plethysmography and single breath helium dilution with CO-diffusion. However, it was not as pronounced as the difference in RV%p, and hence of less importance. When comparing RV and TLC from the different measurement methods, a significant difference was already seen in healthy smokers, and was most probably due to methodological dissimilarities (single breath helium dilution with CO-diffusion measuring only volume communicating with ventilated air space, while body plethysmography also measures trapped air space).

An important aim was to find a lung function parameter that may show early signs of COPD disease, since COPD is an irreversible progressive disease. When diagnosed with COPD today, the disease has already progressed to a partly irreversible limitation in airflow. It is therefore important to identify patients at an earlier stage, so that novel therapies for earlier disease progression can be developed. It is thus also important to study the initial changes in COPD leading to severe stages. Interesting findings in the present study were increases in RV_BP_%p, RV_SB_%p, TLC_BP_%p, TLC_SB_%p, FRC%p, and VA%p already in GOLD1, with the increase in VA%p subsequently resulting in a parallel decrease in DLCO/VA %p. This could be the first signs of inadequate elasticity in GOLD1, resulting in increased lung volumes but sustained flow-volume parameters.

All lung function parameters were affected with an increasing pattern through GOLD1–4, but overall there are only minor differences between healthy smokers and GOLD1. In contrast, there are marked effects in GOLD3-4, while the patients in GOLD2 show a more variable pattern, presenting a heterogeneous group of patient with overlapping lung function results similar to both GOLD1 and GOLD3. This was most clearly seen for Fres, RV_BP_%p–RV_SB_%p, and TLC_BP_%p–TLC_SB_%p (Figures 3–4). The explanation for this is not known, but we can only speculate that the COPD in patients with GOLD1 is possibly due only to chronic bronchitis, while patients with GOLD3-4 have additional emphysema formation. The patients in GOLD2 could be a heterogeneous group of patients with either only chronic bronchitis or in combination with additional emphysema. We aim to investigate this hypothesis further because of the importance to categorize the disease not only by severity but also by disease pattern and phenotype in order to develop more specific therapies.

Another interesting findings were the correlations between several resistance parameters measured by body plethysmography and IOS. These resistance parameters did not relate to lung volume and diffusing capacity parameters suggesting different pathological entities and thereby different COPD phenotypes. Although IOS is an easy method to use, it may not replace spirometry but could be used as a complement or in cases when spirometry cannot be performed. These findings are in accordance with previous speculations on lung diseases overall [20].

The use of a self-filled in quality of life questionnaire is a subjective measure and is questionable as a valuable tool in diagnosing COPD [21]. In the present study there was an increase in CCQ with increasing GOLD stage, and subsequently an overall correlation to all lung function parameters. However, subgrouped within each GOLD stage, there was no correlation between CCQ and any lung function parameter, even though some of the groups were very heterogeneous. The diagnostic use is hence of minor interest but could be valuable in following-up the progress of the disease. It would, however, be interesting to compare the lung function parameters to other markers of disease severity such as 6 minutes walking test, mMRC score, exacerbation frequency, or oxygen saturation to investigate if any lung function parameters correlated better with this than FEV_1_ does. These could possibly then be used to classify disease severity, phenotype the disease, and work as a tool in regulating medication use.

In conclusion, the present study shows that the use of only FEV_1_ in COPD diagnosis and monitoring gives an incomplete characterization of the patients. Extended lung function measurements using body plethysmography, single breath helium dilution with CO-diffusion and IOS show that there was no correlation between FEV_1_, and more advanced lung volume, diffusing capacity, and resistance parameters within different COPD stages. However, other flow-volume parameters, FVC, FEV_1_/FVC, and IC, are related to several more advanced lung function parameters. These parameters should be taken into consideration preferably when the access to more advanced equipment is limited. An interesting parameter is the difference in RV measured by body plethysmography and single breath helium dilution with CO-diffusion that gives a more pronounced measure of air trapping and hyperinflation. Different lung function parameters are of importance in different COPD stages, and a more thorough patient characterization is important for understanding the condition and giving better options for treatment in the future. 

## Figures and Tables

**Table 1:** Patient characteristics.

	Controls	GOLD1	GOLD2	GOLD3	GOLD4
	*n* = 34	*n* = 13	*n* = 22	*n* = 15	*n* = 15
Female/Male, *n*	16/18	6/7	10/12	7/8	9/6
Age, years	67 (66–70)	68 (66–69)	66 (61–68)**	65 (60–69)	66 (62–68)
Smoker/Former smoker, *n*	5/29	7/6	7/15	1/14	0/15
Packyears	27 (21–35)	27 (17–45)	31 (23–51)	40 (30–48)**	35 (28–40)
Body mass index	27 (24–28)	26 (25–28)	27 (24–30)	24 (21–27)	24 (21–27)
No inhaled medication	33	12	7	0	0
SABA use	0	0	7	6	3
LAMA use	0	1	13	15	15
LABA use	1	1	11	11	14
ICS use	1	1	12	13	14
O_2_ use	0	0	0	2	5
CCQ-score	4.0 (1.8–7.0)	6.0 (2.0–10.0)	11.0 (4.0–17.3)***	14.2 (19.0–21.0)^∗∗∗†††‡^	25 (24–30)^∗∗†^
FEV_1_ (L)	2.8 (2.3–3.4)	2.5 (2.2–3.4)	1.9 (1.6–2.2)^∗∗∗††^	1.2 (1.0–1.4)^∗∗∗†††‡‡‡^	0.7 (0.5–0.9)^∗∗∗†††‡‡‡###^
FEV_1_ (%)	95 (90–105)	90 ( 87–94)	61 (55–70)^∗∗∗†††^	41 ( 33–49)^∗∗∗†††‡‡‡^	27 (22–28)^∗∗∗†††‡‡‡###^
FVC (L)	3.7 (3.0–4.3)	4.2 (3.3–4.8)	3.4 (2.9–4.1)	2.9 (2.2–3.4)^∗∗††‡^	2.1 (1.1–3.0)^∗∗∗†††‡‡‡#^
FVC (%)	96 (88–103)	106 (99–114)**	85 (73–94)^∗∗†††^	76 (68–83)^∗∗∗†††‡^	63 (35–73)^∗∗∗†††‡‡‡#^
FEV_1_/FVC	0.77 (0.74–0.80)	0.66 (0.63–0.70)***	0.58 (0.49–0.65)^∗∗∗††^	0.39 (0.36–0.47)^∗∗∗†††‡‡‡^	0.31 (0.30–0.46)^∗∗∗†††‡‡‡#^

*Significant difference compared to healthy smokers, ^†^significant difference compared to GOLD1, ^‡^significant difference compared to GOLD2, ^#^significant difference compared to GOLD3, one symbol flagging *P* < 0.05, two symbols flagging *P* < 0.01 and three symbols flagging *P* < 0.001. SABA: short acting beta agonist, LAMA: long acting muscarinic agonist, LABA: long acting beta agonist, ICS: inhaled corticosteroids, O_2_: oxygen therapy. All data are presented as median (interquartile range) or otherwise stated.

**Table 2:** Body plethysmography and single breath helium dilution with CO-diffusion (SB) parameters.

	Controls	GOLD1	GOLD2	GOLD3	GOLD4
*Body plethysmography (BP) *					
*R*_in_, cmH_2_O∗s/L	2.0 (1.6–2.4)	2.0 (1.6–2.8)	3.0 (2.1–3.2)**	3.6 (2.8–5.3)^∗∗∗†††‡‡^	35.4 (4.6–6.6)^∗∗∗††† ‡‡‡##^
*R*_ex_, cmH_2_O∗s/L	3.2 (2.4–3.7)	2.7 (2.3–4.4)	4.8 (3.2–6.8)^∗∗††^	13.2 (5.1–21.0)^∗∗∗†††‡‡^	21.2 (14.1–33.1)^∗∗∗†††‡‡‡#^
IC, L	3.2 (2.7–3.8)	3.0 (2.7–3.7)	2.7 (2.4–3.2)	2.3 (1.9–3.0)^∗∗∗††^	1.4 (1.0–2.6)^∗∗∗†††‡‡^
IC, %p	101 (88–108)	97 (88–108)	85 (73–98)^†^	77 (67–91)^∗∗∗††^	52 (31.66)^∗∗∗†††‡‡##^
RV_BP_, L	2.5 (2.2–2.9)	2.5 (2.3–3.0)	2.8 (2.4–3.1)	3.6 (3.2–4.8)^∗∗∗†††‡‡‡^	4.5 (4.3–5.3)^∗∗∗†††‡‡‡#^
RV_BP_, %p	111 (98–120)	115 (105–124)	124 (100–144)	174 (148–187)^∗∗∗†††‡‡‡^	217 (193–245)^∗∗∗†††‡‡‡##^
TLC_BP_, L	6.4 (5.4–7.3)	6.7 (5.7–8.1)	6.2 (5.7–7.1)	7.3 (6.0–7.7)	7.0 (5.7–7.6)
TLC_BP_, %p	105 (97–111)	108 (107–117)*	104 (88–123)	113 (107–126)**	121 (100–138)^∗∗‡^
FRC_BP_, L	3.1 (2.6–3.4)	3.4 (3.0–4.1)*	3.6 (3.2–4.0)**	4.4 (3.9–5.7)^∗∗∗†‡‡^	5.4 (4.6–5.8)^∗∗∗†††‡‡‡^
FRC_BP_, %p	94 (88–108)	109 (102–120)**	106 (91–142)*	135 (123–152)^∗∗∗†††‡‡^	172 (161–203)^∗∗∗†††‡‡‡##^

*Single breath helium dilution with carbon monoxide diffusion (SB) *					
DLCO_SB_, mmol/min/kPa	6.2 (5.3–7.1)	5.6 (4.5–7.7)	5.2 (4.6–6.3)**	3.0 (2.4–4.5)^∗∗∗†††‡‡‡^	1.8 (1.1–2.5)^∗∗∗†††‡‡‡###^
DLCO_SB_, %p	75 (69–83)	75 (53–87)	63 (53–70)***	40 (32–46)^∗∗∗†††‡‡‡^	22 (15–29)^∗∗∗†††‡‡‡###^
VA, L	5.3 (4.7–5.3)	5.6 (4.9–5.6)	4.8 (4.2–5.7)^†^	4.4 (4.0–5.2)^∗††^	3.7 (3.1–4.5)^∗∗∗†††‡‡‡^
VA, %p	90 (83–97)	96 (93–103)*	84 (74–89)^∗†††^	78 (64–83)^∗∗∗†††^	67 (58–77)^∗∗∗†††‡‡‡^
DLCO_SB_/VA, mmol/min/kPa/L	1.2 (1.1–1.3)	1.1 (0.9–1.2)*	1.1 (0.9–1.3)*	0.71 (0.64–0.83)^∗∗∗†††‡‡‡^	0.46 (0.35–0.56)^∗∗∗†††‡‡‡###^
DLCO_SB_/VA, %p	89 (78–95)	73 (61–90)*	76 (64–93)*	50 (45–60)^∗∗∗††‡‡‡^	35 (23–41)^∗∗∗†††‡‡‡###^
RV_SB_, L	1.9 (1.7–2.0)	2.0 (1.6–2.3)	1.7 (1.5–2.0)^†^	1.5 (1.3–1.9)^∗∗†^	1.6 (1.3–1.8)*
RV_SB_, %p	81 (72–87)	90 (77–96)	77 (64–89)	67 (59–79)^∗†^	69 (58–95)
TLC_SB_, L	5.5 (4.9–6.2)	5.8 (5.1–7.1)	5.0 (4.4–5.9)^†^	4.6 (4.1–5.3)^∗††^	3.9 (3.3–4.7)^∗∗∗†††‡‡‡^
TLC_SB_, %p	91 (85–97)	97 (93–103)*	85 (75–90)^∗†††^	79 (65–84)^∗∗∗†††^	68 (60–77)^∗∗∗†††‡‡‡^
FRC_SB_, L	2.5 (2.1–2.7)	3.0 (2.8–3.2)**	2.5 (2.1–2.8)^†^	2.1 (1.6–2.9)^†^	2.4 (1.8–2.9)^†††‡‡‡^
FRC_SB_, %p	75 (67–87)	93 (81–104)**	76 (66–91)^††^	71 (56–80)^††^	69 (61–102)^†††‡‡‡^

*Difference between BP and SB *					
RV %p_BP−SB_	28 (19–40)	33 (23–40)	43 (29–62)^∗†^	95 (80–129)^∗∗∗†††‡‡‡^	149 (119–192)^∗∗∗†††‡‡‡##^
TLC %p_BP−SB_	14 (11–18)	14 (11–18)	18 (13–27)*	35 (30–49)^∗∗∗†††‡‡‡^	57 (39–70)^∗∗∗†††‡‡‡#^

*Significant difference compared to healthy smokers, ^†^significant difference compared to GOLD1, ^‡^significant difference compared to GOLD2, ^#^significant difference compared to GOLD3, one symbol flagging *P* < 0.05, two symbols flagging *P* < 0.01 and three symbols flagging *P* < 0.001. All data are presented as median (interquartile range).

**Table 3:** Impulse oscillometry parameters.

	Controls	GOLD1	GOLD2	GOLD3	GOLD4
*R*_5_, kPa∗/L	0.27 (0.23–0.32)	0.29 (0.26–0.31)	0.37 (0.30–0.44)^∗∗∗†^	0.50 (0.39–0.67)^∗∗∗†††‡‡^	0.52 (0.41–0.70)^∗∗∗†††‡‡‡^
*R*_5_ %p	90 (68–91)	83 (74–97)	105 (90–120)^∗∗∗†^	136 (121–195)^∗∗∗†††‡‡‡^	134 (126–173)^∗∗∗†††‡‡‡^
*R*_20_, kPa∗/L	0.21 (0.18–0.26)	0.22 (0.19–0.27)	0.26 (0.20–0.28)	0.30 (0.24–0.38)^∗∗∗††‡^	0.28 (0.25–0.34)^∗∗†^
*R*_20_ %p	70 (62–89)	79 (64–86)	81 (73–96)*	104 (85–130)^∗∗∗†††‡‡^	89 (79–99)^∗∗†^
*R*_5_–*R*_20_, kPa∗/L	0.04 (0.03–0.08)	0.07 (0.03–0.10)	0.12 (0.06–0.15)^∗∗∗†^	0.17 (0.12–0.33)^∗∗∗†††‡‡^	0.24 (0.17–0.36)^∗∗∗†††‡‡‡^
*R*_5_–*R*_20_ %p	100 (67–150)	167 (75–192)	250 (131–306)^∗∗∗†^	388 (281–554)^∗∗∗†††‡‡^	425 (367–650)^∗∗∗†††‡‡‡^
AX, kPa∗/L	0.18 (0.13–0.44)	0.16 (0.11–0.57)	0.69 (0.34–1.49)^∗∗∗††^	1.64 (0.97–3.61)^∗∗∗†††‡‡^	3.17 (1.46–3.54)^∗∗∗†††‡‡‡^
*F*_res_, Hz	10.5 (8.9–14.6)	12.5 (9.1–15.5)	16.4 (13.9–19.9)^∗∗∗††^	20.4 (18.2–25.3)^∗∗∗†††‡^	23.9 (21.3–27.7)^∗∗∗†††‡‡‡#^
X5, kPa∗/L	−0.09 (−0.11–−0.07)	−0.08 (−0.12–−0.06)	−0.14 (−0.22–−0.10)^∗∗†^	−0.25 (−0.43–−0.16)^∗∗∗†††‡‡^	−0.42 (−0.49–−0.23)^∗∗∗†††‡‡‡^
X5 %p	199 (104–312)	175 (145–263)	389 (182 –541)^∗∗∗†^	494 (447–795)^∗∗∗†††^	677 (501–859)^∗∗∗†††‡‡^

*Significant difference compared to healthy smokers, ^†^significant difference compared to GOLD1, ^‡^significant difference compared to GOLD2, ^#^significant difference compared to GOLD3, one symbol flagging *P* < 0.05, two symbols flagging *P* < 0.01 and three symbols flagging *P* < 0.001. All data are presented as median (interquartile range).

**Table 4:** Correlations between established flow-volume parameters and extended volume and resistance parameters.

	Volume		Resistance			
	RV%p_BP-SB_	TLC%p_BP-SB_	*R*_in_	*R*_ex_	*R*_5_–*R*_20_	*F*_res_
*Flow-volume *						
FEV_1_%p						
Controls	0.15	0.31	−0.01	0.06	−0.12	−0.21
GOLD1	0.50	−0.25	−0.18	−0.26	−0.27	−0.36
GOLD2	−0.37	−0.34	−0.19	− 0.20	−0.26	−0.18
GOLD3	−0.21	0.12	−0.16	0.18	−0.3	−0.32
GOLD4	−0.33	−0.34	−0.14	−0.12	−0.14	−0.11
FVC%p						
Controls	0.14	0.34	0.12	0.14	−0.05	−0.07
GOLD1	0.14	0.15	−0.19	−0.16	−0.22	−0.32
GOLD2	0.59**	0.60**	−0.35	−0.29	−0.29	−0.14
GOLD3	−0.14	0.01	−0.31	−0.05	−0.22	−0.2
GOLD4	−0.55*	−0.21	−0.33	−0.45	−0.26	−0.11
FEV_1_/FVC						
Controls	−0.02	−0.22	−0.26	−0.11	−0.19	−0.25
GOLD1	0.34	−0.07	−0.13	−0.29	−0.15	−0.13
GOLD2	−0.71***	−0.71***	0.12	0.09	0.15	0.08
GOLD3	−0.31	0.04	−0.08	0.35	−0.06	−0.14
GOLD4	0.69**	0.37	0.32	0.58*	0.24	0.05
IC%p						
Controls	0.30	0.42∗	0.07	−0.02	−0.06	−0.04
GOLD1	0.29	−0.04	0.61*	0.73**	−0.06	0.09
GOLD2	0.66***	0.63**	0.06	0.29	0.11	0.10
GOLD3	0.11	0.46	−0.24	0.35	−0.3	−0.42
GOLD4	0.72**	0.01	−0.36	−0.61*	−0.61	−0.13

*Resistance *						
*R*_5_–*R*_20_						
Controls	−0.05	−0.12	0.43**	0.37*	—	0.85***
GOLD1	−0.26	0.00	0.44	0.21	—	0.94***
GOLD2	0.08	0.03	0.80***	0.88***	—	0.87***
GOLD3	0.04	0.22	0.64*	0.44	—	0.86***
GOLD4	0.29	0.54*	0.71**	0.55	—	0.69**
*F*_res_						
Controls	−0.04	−0.2	0.47**	0.40*	0.85***	—
GOLD1	−0.28	0.27	0.61*	0.4	0.94***	—
GOLD2	0.08	0.02	0.63**	0.77***	0.87***	—
GOLD3	0.13	0.26	0.50	0.41	0.86***	—
GOLD4	−0.05	0.13	0.61*	0.47	0.69**	—

Data are presented as *r*-values. *P < 0.05, **P < 0.01 ***P < 0.001.

## Abbreviations

BP: Body plethysmography
CCQ: Clinical COPD Questionnaire
COPD: Chronic pulmonary obstructive disease
GOLD: Global initiative of obstructive lung diseases
FEV_1_: Forced expiratory volume in 1 s
FRC: Functional residual capacity
FVC: Forced vital capacity
IC: Inspiratory capacity
*R*_ex_: Expiratory resistance
*R*_in_: Inspiratory resistance
RV: Residual volume
TLC: Total lung capacity
DLCO: Diffusing capacity of the lung for carbon monoxide
VA: Alveolar volume.
